# Association between Late-Eating Pattern and Higher Consumption of Ultra-Processed Food among Italian Adults: Findings from the INHES Study

**DOI:** 10.3390/nu15061497

**Published:** 2023-03-20

**Authors:** Marialaura Bonaccio, Emilia Ruggiero, Augusto Di Castelnuovo, Claudia Francisca Martínez, Simona Esposito, Simona Costanzo, Chiara Cerletti, Maria Benedetta Donati, Giovanni de Gaetano, Licia Iacoviello

**Affiliations:** 1 Department of Epidemiology and Prevention, IRCCS NEUROMED, 86077 Pozzilli, Italy; 2 Mediterranea Cardiocentro, 80122 Napoli, Italy; 3 Department of Medicine and Surgery, Research Center in Epidemiology and Preventive Medicine (EPIMED), University of Insubria, 21100 Varese, Italy

**Keywords:** meal timing, late eating, food processing, ultra-processed food, NOVA classification

## Abstract

Late eating is reportedly associated with adverse metabolic health, possibly through poor diet quality. We tested the hypothesis that meal timing could also be linked to food processing, an independent predictor of health outcomes. We analysed data on 8688 Italians (aged > 19years) from the Italian Nutrition & HEalth Survey (INHES) established in 2010–2013 throughout Italy. Dietary data were collected through a single 24 h dietary recall, and the NOVA classification was used to categorize foods according to increasing levels of processing: (1) minimally processed foods (e.g., fruits); (2) culinary ingredients (e.g., butter); (3) processed foods (e.g., canned fish); (4) ultra-processed foods (UPFs; e.g., carbonated drinks, processed meat). We then calculated the proportion (%) of each NOVA group on the total weight of food eaten (g/d) by creating a weight ratio. Subjects were classified as early or late eaters based on the population’s median timing for breakfast, lunch and dinner. In multivariable-adjusted regression models, late eaters reported a lower intake of minimally processed food (β = −1.23; 95% CI −1.75 to −0.71), a higher intake of UPF (β = 0.93; 0.60 to 1.25) and reduced adherence to a Mediterranean Diet (β = −0.07; −0.12 to −0.03) as compared to early eaters. Future studies are warranted to examine whether increased UPF consumption may underpin the associations of late eating with adverse metabolic health reported in prior cohorts.

## 1. Introduction

Obesity and associated cardiometabolic diseases continue to rise worldwide despite extensive public health efforts to reverse this trend [1]. Unhealthy diets, i.e., diets not meeting nutritional requirements, are major risk factors for obesity and associated diseases [2,3], and therefore, common strategies to tackle obesity and diet-related diseases have been almost exclusively focused on food composition, leading to recommendations to reduce sugar, salt and fat while emphasizing high intakes of foods that are natural sources of fibre, vitamins and minerals [4].

Among the factors that possibly contribute to the rise in obesity and cardiometabolic diseases, growing attention has been paid to the timing of food intake (i.e., the time when meals are usually consumed), which has been associated with various indicators of adiposity, possibly, but not entirely, through higher energy intake [5,6,7,8,9,10,11,12].

Population studies suggest that late eating, which refers to a delay in the timing of meals (usually the main meal of the day or the last meal, i.e., dinner) [12] may be a factor implicated in obesity and other non-communicable diseases related to nutrition [13,14,15]. Potential mechanistic links through which meal timing may promote obesity and associated diseases include, among others, the lower diet quality and higher calorie intake observed in late eaters [16,17,18]. However, no prior studies to date have evaluated the possible association of meal timing with the intake of foods with different degrees of processing. Actually, it has been suggested that obesity prevalence continues to increase concomitantly with the increased consumption of ultra-processed foods (UPFs) [19]. According to the NOVA classification, UPFs are industrial formulations of ingredients, containing little or no whole food and typically including flavouring and colouring agents, emulsifiers and other cosmetic additives [20]. Consistently, population-based cohorts support a direct association of a large dietary share of UPFs with obesity [21,22] and cardiometabolic diseases [23], as well as with the incidence of major chronic diseases, regardless of the overall diet quality [24].

To fill this knowledge gap, we tested the hypothesis that the meal timing pattern is differentially associated with the intake of foods that have different food processing levels according to the NOVA classification. This study was conducted using a large dataset of adults recruited throughout Italy in 2010–2013.

## 2. Materials and Methods

### 2.1. Study Population

The data are from the Italian Nutrition & HEalth Survey (INHES), which was a 3-year telephone-based survey on nutrition and health designed to collect information on dietary habits (i.e., quality, quantity, food and meal patterns), food choice determinants, and food health awareness of the Italian population according to geographical distribution, age, gender and socioeconomic status. A total of 9422 men and women aged ≥4 years throughout Italy were enrolled between November 2010 and November 2013. Details about this cohort have been previously described [25].

To capture an adequate proportion of weekdays and weekends, a survey calendar was organized at a group level accordingly in order to distribute the sample subjects across four seasons (excluding Christmas, Easter and mid-August periods).

During the recruitment phase, the computer-assisted telephone interview method was used to collect dietary data (dietary habits and behaviour), the health status of the subjects, risk factors, anthropometric measurements (for example, height and weight) and health perception. Given the study objective, participants were excluded for the following reasons: subjects below 20 years of age (n = 571), missing data on diet (n = 2), extreme energy intakes reported (<800 kcal/d in men and <500 kcal/d in women or >4000 kcal/d in men and >3500 kcal/d in women; n = 159) and missing data on meal timing (n= 2). Therefore, a total of 8688 subjects were analysed.

### 2.2. Assessment of Dietary Data

A self-recorded diary, using computer-based 1-day 24-h dietary recall interview (24-HDR) software, and an Italian version of the European Food Propensity Questionnaire were used to record dietary data [26,27].

Subjects were instructed to recall and record the following data for each meal consumed: (a) time and place of food intake; (b) detailed description of foods (or beverages) and (c) the quantity of intake and the food brand chosen (for manufactured foods). Further, a picture booklet was used as a reference by the subjects to report portion sizes. Lastly, participants answered whether they were currently on any diet and whether their consumption differed from their habitual diet.

Individual food items and recipes reported by the participants were later matched with those available in the food list of the data management system INRAN-DIARIO 3.1 [26,28] by a nutritionist during the interviews.

Finally, a total of 2000 single food items extracted from the final output database were included in the software food list.

The NOVA classification [29] was used to categorize each food item into one of the following categories according to the extent and purpose of food processing: (1) fresh or minimally processed foods (e.g., fruit, meat, milk); (2) processed culinary ingredients (e.g., oils, butter, sugar); (3) processed food items (e.g., canned fish, unpackaged freshly made breads); or (4) UPFs containing predominantly industrial substances and little or no whole foods (e.g., carbonated drinks, processed meat, sweet or savoury packaged snacks). Consumption (in g/d) in each of the four NOVA groups and the percentage they represented with respect to the total amount of food eaten were determined in order to obtain a weight ratio. We used this approach instead of the energy ratio because total food amounts better account for non-nutritional factors related to food processing (e.g., neo-formed contaminants, additives and alterations to the structure of raw foods) [30]. The full list of individual foods and food groups categorized according to the NOVA classification is available in Table 1. For analyses on individual meal types, we calculated the consumption in each NOVA group separately for breakfast, lunch and dinner. Adherence to the Mediterranean Diet was evaluated by the Mediterranean Diet Score (MDS) as proposed by Trichopoulou et al. [31]. Briefly, we assigned 1 point to healthy foods (i.e., fruits and nuts, vegetables, legumes, fish, cereals, monounsaturated-to-saturated fat ratio) whose consumption was above the sex-specific medians of intake in the adult population of the whole INHES cohort; foods presumed to be detrimental (i.e., meat and dairy products) were given a positive score if their consumption was below the median. All other intakes received 0 points. For alcohol intake (ethanol), participants who consumed alcohol (men: 10–50 g/d; women: 5–25 g/d) scored 1 point; otherwise, the score was 0. The Mediterranean Diet Score potentially ranges from 0 to 9 (the latter reflecting maximum adherence).

To evaluate overall diet quality, we also calculated the Food Standards Agency Nutrient Profiling System (FSAm-NPS) dietary index, which is used to compute the Nutri-Score front-of-pack labelling system that ranks food items according to their nutritional value [32].

The FSAm-NPS score was calculated as previously implemented in other population cohorts [24,33] as follows: for all foods and beverages consumed, based on composition for each 100 g of content, 0 to 40 points were allocated for nutrients that should be consumed in limited amounts (A points), i.e., total sugars (g), saturated fats (g), sodium (mg) and energy (kJ), and 0 to 15 points were given for nutrients or components that should be promoted, i.e., dietary fibre (g) and protein (g), and for fruit, vegetables, legumes and nuts (%) (C points). The total score of the product was calculated by subtracting the sum of C points from the sum of A points. Thus, the final FSAm-NPS score for each food/beverage was based on a scale that could theoretically range from −15 (healthiest food) to +40 (least healthy food). Based on this overall FSAm-NPS score, the Nutri-Score labelling system categorizes food products into five colours, associated with letters A (dark green) to E (dark orange), reflecting their nutritional quality [32]. The FSAm-NPS dietary index (DI) was computed at the individual level as an energy-weighted mean of the FSAm-NPS scores of all foods and beverages consumed by each participant using the following equation:

$$\mathrm{FSA}-\mathrm{NPS}\;\mathrm{DI}\;=\frac{\sum_{i\;=1}^{n}{\mathrm{FS}}_{i}E_{i}}{\sum_{i\;=1}^{n}E_{i}}$$

FS_i_ represents the score of food/beverage ‘i’, E_i_ is the energy intake from food/beverage ‘i’ specific to each participant, and ‘n’ is the total number of foods/beverages consumed. An increase in the FSAm-NPS dietary index values therefore reflects a decrease in the overall diet quality value.

### 2.3. Assessment of Meal Timing

The timing of main meals (i.e., breakfast, lunch and dinner) was obtained by using information provided by participants during the 24 h dietary recall, where they were asked to indicate the time of each eating occasion. For each main meal, we calculated the study population sample’s median time and assigned 1 point to those participants reporting having (a) breakfast after 7 am (study sample median time); (b) lunch after 1 p.m. (study sample median time); and (c) dinner after 8 p.m. (study sample median time). Individuals consuming meals before the median time were given 0 points. Participants scoring ≥2 points were considered to have a late meal timing pattern; otherwise, people were classified as having an early meal timing pattern. For simplification, we called them late eaters and early eaters, respectively.

### 2.4. Ascertainment of Covariates

Education was based on the highest qualification attained and was categorized as up to elementary school (corresponding to ≤5 years of study), lower secondary (>5–≤8 years), upper secondary (>8–≤13 years) and postsecondary (>13 years). Present occupations were categorized into six groups: manual, non-manual, housewife, retired, student and unemployed. Marital status was defined as married/living in a couple, single, separated/divorced and widowed. The definition of urban or rural environments was based on the urbanization level described by the European Institute of Statistics (EUROSTAT definition)—obtained by the tool ‘Atlante Statistico dei Comuni’ provided by the Italian National Institute of Statistics [34]. Subjects were classified as never (one who has never smoked, or who has smoked less than 100 cigarettes in the lifetime), current (smoking one or more cigarettes per day at the time of the interview), former (one who had quit smoking at the time of interview) or occasional smokers (smoking less than 1 cigarette per day at the time of interview). History of cardiovascular disease and cancer and a previous diagnosis of diabetes, hyperlipidaemia or hypertension were self-reported and categorized as yes/no. Body mass index (BMI) was calculated by using self-reported measurements of height and weight, calculated as kg/m^2^ and grouped into three categories: normal (≤25 kg/m^2^), overweight (>25–<30 kg/m^2^) or obese (≥30 kg/m^2^). Self-reported sport activity was used as a categorical variable (yes/no).

### 2.5. Statistical Analysis

The general characteristics of the analytic sample according to early and late-eating patterns are presented as numbers and percentages for categorical variables and means with standard deviations (SDs) for continuous traits. Differences in the distribution of baseline covariates were calculated using generalized linear models adjusted for age, sex and energy intake (GENMOD procedure for categorical variables and GLM procedure for continuous variables in SAS software).

Beta coefficients with 95% confidence intervals (95% CI) from multivariable-adjusted linear regression analyses were used to evaluate the association between the meal timing pattern (independent variable) and each category of NOVA (continuous dependent variable) or dietary index (i.e., the Mediterranean Diet Score and the FSAm-NPS dietary index; continuous dependent variables). Each dietary variable was standardized to one standard deviation to allow comparison. An *a priori* approach was used to select potential covariates instead of statistical criteria [35]. Two models were ultimately fitted: model 1 was adjusted for age, sex and energy intake, and multivariable model 2 was model 1 but further adjusted for education, geographical area, place of residence, sport activity, occupation, marital status, smoking, BMI, cardiovascular disease, cancer, hypertension, diabetes and hyperlipidaemia. To maximize data availability, missing data on covariates were handled using multiple imputation (SAS PROC MI, followed by PROC MIANALYZE; n = 10 imputed datasets).

We conducted subgroup analyses to test the robustness of the findings by analysing the potential effect modification of the association of the meal timing pattern with each dietary score by various risk factors, such as age (19–50 years; 51–65 years and 66–97 years) and sex. We used SAS/STAT software, version 9.4 (SAS Institute Inc., Cary, NC, USA), for the analysis.

## 3. Results

The analytic sample consists of 4053 men (46.7%) and 4635 women (53.3%) with a mean age of 56.9 years (±14.6). The average (SD) weight contributions of unprocessed/minimally processed foods, culinary ingredients, processed foods and UPFs to the diet were 73.7% (±12.0), 2.6% (±1.2), 15.9% (±10.7) and 7.8% (±7.0), respectively. More than half (58.1%) of the total calories came from unprocessed/minimally processed foods and culinary ingredients, while 24.6% came from processed food, and 17.3% were from UPFs.

The characteristics of the study participants according to the meal timing pattern are presented in Table 2. As compared to early eaters, late eaters were younger, were more likely to live in Southern Italy and urban environments, had a higher educational level and were prevalently non-manual workers. Additionally, late eaters were less likely to report chronic diseases (e.g., CVD) or other health conditions (e.g., hypertension and hyperlipidaemia). No relevant differences in BMI, diabetes or history of cancer were found. Differences in dietary factors were also observed between meal timing patterns. Specifically, late eaters tended to consume less energy from carbohydrates while reporting higher energy from fats (Table 3).

In multivariable-adjusted regression analyses, we found that late eaters were less likely to consume unprocessed/minimally processed foods as compared to early eaters (β = −0.10; 95% CI −0.14 to −0.06) while reporting the increased consumption of UPFs (β = 0.13; 95% CI 0.09 to 0.18) and processed culinary ingredients (β = 0.05; 95% CI 0.01 to 0.10); eating late was also found to be inversely associated with adherence to the Mediterranean Diet (β = −0.07; 95% CI −0.12 to −0.03) and directly associated with the FSAm-NPS dietary index (β = 0.10; 95% CI 0.05 to 0.14) (Table 4; Model 2). The direction and strengths of these associations were substantially confirmed in all age groups and in men and women, especially for UPF consumption and diet quality indices (Supplementary Tables S1 and S2); however, the relationships of late eating with unprocessed/minimally processed food or processed food intake were stronger in the young group than in the elderly (Supplementary Table S1). Additionally, an effect modification by sex was observed in relation to the consumption of unprocessed/minimally processed foods and culinary ingredients (Supplementary Table S2).

**Figure 1:**
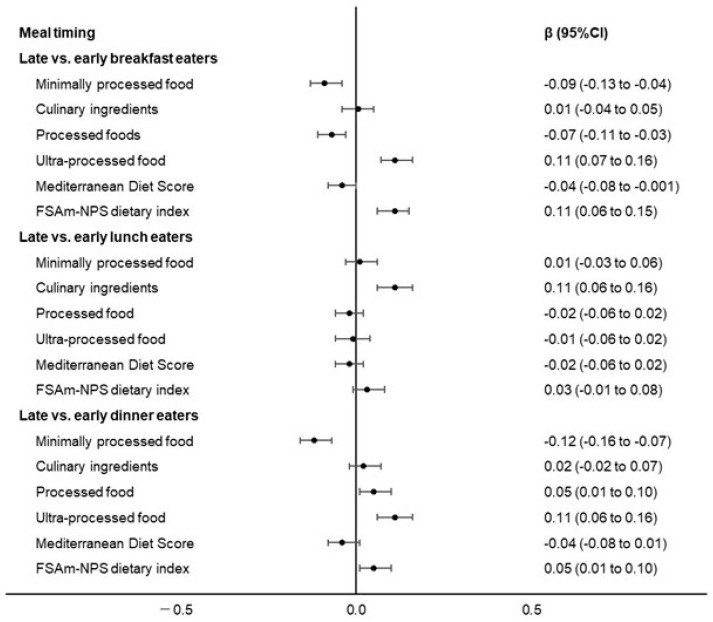
Timing of food intake for individual meals (late vs. early eaters) associated with food processing according to NOVA classification, adherence to the Mediterranean Diet and the Food Standards Agency Nutrient Profiling System (FSAm-NPS) dietary index in 8688 participants (20–97 years) from the INHES study, Italy, 2010–2013. Regression coefficients β with 95% CIs from a multivariable-adjusted linear regression including age, sex, energy intake, place of residence, educational level, occupation, marital status, smoking status, sport activity, body mass index, history of cardiovascular disease, history of cancer, diabetes, hyperlipidaemia and hypertension. Each dietary variable was standardized to allow comparison.

Analyses separated by meal type showed that late breakfast eating was associated with the reduced consumption of unprocessed/minimally processed foods and processed foods and a higher intake of UPFs at breakfast, as well as with lower adherence to the Mediterranean Diet and a higher FSAm-NPS dietary index. Similarly, participants who had delayed dinners were more likely to eat processed foods or UPFs and tended to reduce the intake of unprocessed/minimally processed foods, and also reported less adherence to a Mediterranean Diet and a larger dietary share of foods with poor nutritional quality. Finally, late lunch eaters reported a higher intake of processed culinary ingredients (Figure 1).

## 4. Discussion

In this large cohort of 8688 adults from the general Italian population, a late-eating pattern was associated with both a higher consumption of UPFs and a lower intake of unprocessed/minimally processed foods, as well as with poorer diet quality. Evidence from population studies has consistently suggested that the timing of meal intake is a reliable predictor of cardiometabolic health outcomes, with late eating being reportedly associated with obesity and glucose intolerance in observational studies [10,36]. The key role of timed meals has been also supported by animal [37] and intervention studies in humans showing that late eating may adversely impact the success of weight-loss therapy [38].

Mechanistic hypotheses to support the association of late eating with adverse cardiometabolic health are likely multifactorial and include the fact that late eating may contribute to circadian misalignment, i.e., a lack of synchrony of light/dark cycles and behavioural rhythms with the endogenous circadian system [38,39,40], which was found to adversely impact both energy balance and glycaemic control [41] and changes in the diversity of the microbiota [42].

A number of studies indicate that late eaters tend to have a lower overall diet quality and higher energy intake [16,17,43,44], which may in part explain the adverse cardiometabolic health associated with delaying meals to later in the day; this was also confirmed by our analyses showing that late eating was associated with reduced adherence to a traditional Mediterranean Diet and higher values of the FSAm-NPS dietary index, which is used to compute the Nutri-Score front-of-pack labels and reflects the consumption of less-nutrient-dense foods. However, others reported that energy intake and overall diet quality were not found to vary significantly across eating times [39].

As all prior studies were focused on the nutritional composition of diets, regardless of food processing levels, we used a complementary approach by examining whether meal timing is differentially associated with the food intakes with different levels of processing according to the NOVA classification.

UPF intake is on arise worldwide and constitutes more than half of the total calories eaten in the US, UK and Canada [45,46,47] while being less consumed in Mediterranean countries, such as Italy [48] and Spain [49]. An increasing number of large-scale population studies indicate that elevated intakes of UPFs can be a major threat to human health, being directly associated with an increased risk of cardiovascular disease, cancer and diabetes, as well as reduced survival [23,24]. A systematic review summarizing the evidence for the association between food processing and cardiometabolic factors in adults found that a large dietary share of UPFs is positively associated with worse cardiometabolic health, as reflected by increased levels of overweight and obesity, metabolic syndrome and high blood pressure [50]. Additionally, a high proportion of UPFs in the diet was linked to altered levels of inflammation [51], which was found to be increased in association with mistimed meals in both animals [52] and humans [53].

Both the direct association of the meal timing pattern with UPFs and its inverse relationship with unprocessed/minimally processed foods observed in our study suggest that the degree of food processing could be among the potential mechanisms/factors that link mistimed meals to impaired cardiometabolic outcomes. Besides being nutrient-poor (e.g., rich in fat, sodium and salt, and low in fibre and nutrients), UPFs are a major dietary source of chemicals (e.g., endocrine-disrupting chemicals such as bisphenol and phthalates commonly used in food packaging) and neo-formed compounds (e.g., acrylamide), which may have severe implications for health, as suggested by robust research, ranging from laboratory-based to prospective epidemiological studies [54].

Most importantly, food processing impacts both the nutritional composition (e.g., decreased antioxidant potential of some foods resulting from removing germ and bran) and food matrix (i.e., the ‘architecture’ of the food, which derives from nutrient interactions), which is crucial to the food’s overall health potential, specifically in satiety and glycaemic responses, as well as in determining nutrient bioavailability [55].

While complex, natural, minimally or unprocessed foods have more or less intact structures, and their nutritional properties are substantially unaltered [55], highly processed foods are typically unstructured, fractionated and usually heavily supplemented with free glucose and sucrose, which renders glucose more available for absorption, thereby increasing blood glycaemic response [56]. Diets with a large share of foods with a high glycaemic index are well-established risk factors for cardiometabolic diseases and mortality [57].

Interestingly, in our study late eating was associated with an approximately absolute 1% higher proportion of UPF intake relative to the total food eaten; prior cohort studies showed that even such a small increment possibly leads to a higher risk of mortality both in general populations [24] and among people with pre-existing cardiovascular disease [58]. Despite consuming more UPFs, late eaters also tended to report lower diet quality overall, and in this regard, it is worth noting that most highly processed foods are typically less nutrient-dense [59]. In addition, diets high in UPFs were found to have a higher impact on mortality than the overall diet quality [24].

Lastly, a late meal pattern in our study was associated with younger age, a higher educational level and being single; all these characteristics were reportedly associated with a higher consumption of UPFs in previous cohort studies [48,60], while unmarried individuals were also found to have lower diet quality overall [61,62]. However, our estimates were from multivariable-adjusted models that also account for these socioeconomic and demographic factors, and other drivers for UPF consumption need consideration (e.g., heavy marketing, availability, low cost, attractiveness, high palatability and domination of food supply chains) [20].

### Strengths and Limitations

To the best of our knowledge, this is the first study that analysed meal timing in association with food processing and also with the dietary index underpinning the Nutri-Score front-of-pack label. The major strengths of this study include a large sample size representative of the Italian population, with a complete assessment of diet, lifestyle and other covariates used to minimize, at least in part, confounding. Moreover, the use of 24 h recall is more advantageous than other tools (e.g., food frequency questionnaires) to assess participants’ diets and to classify foods based on the extent of processing according to NOVA [63]. Despite its strengths, among its limitations, we acknowledge the observational nature of our study and the cross-sectional design of the analyses, which limits causal inference. Further, errors in the visual display of foods and potential bias could have been introduced by the interviewer in the telephone-based survey. Additionally, the decline in the use of landline phones may have resulted in an under-representation of respondents. Another weakness is that the study relied on self-reported dietary data, which are susceptible to bias and error, including social desirability and recall bias, imprecision in assessing portion sizes and inadequacies in food composition tables; however, data were collected by trained interviewers, and each participant received by mail, beforehand, a short photograph atlas and guidance notes to estimate food portion sizes. It was not possible to include some unmeasured factors as confounders due to their unavailability; however, it is a weakness in any observational study. Limitations also include that we dichotomized our population into early and late eaters using the population median timing, as a consensus on the most suitable approach to quantifying food timing is still lacking [39]. We also acknowledge that the NOVA classification remains controversial, mainly due to its equivocal definition of ultra-processed food and multiple revisions and refinements over time [64]; however, its utility value in nutrition epidemiology research has been widely acknowledged allowing comparison with previous studies. Finally, the generalizability of our findings might be limited to the Italian population.

## 5. Conclusions

As well as reporting poor diet quality overall, late eaters are prone to consume more UPFs and fewer minimally processed food than early eaters. These findings contribute to increased knowledge on the mechanisms underpinning the association between late eating and adverse cardiometabolic health previously reported in several experimental and observational studies [12,13,39]. Anticipating the timing of meals may provide a complementary strategy for reducing UPF consumption and increasing unprocessed or minimally processed food intakes, which typically require more time and effort than ready-to-eat/heat meals. Undeniably, mistimed meals are strongly influenced by several factors, especially socioeconomic conditions that are difficult to tackle. Further research is warranted to test whether the consumption of UPFs could be a mediator of the association between mistimed meals and adverse cardiometabolic health.

## Figures and Tables

**Table 1:** Classification of individual food items and food groups by degree of food processing according to NOVA in the INHES study, Italy, 2010–2013.

NOVA Food Category	Food Items
Group 1: Unprocessed or minimally processed foods	Water; fresh, squeezed or dried fruits and leafy and root vegetables; nuts; fresh legumes; wheat; rice; pasta; flour; potatoes; meat; poultry; fish and seafood; milk; plain yogurts without added sugar; eggs; spices; tea and coffee.
Group 2: Processed culinary ingredients	Vinegars; creams; vegetable oils; butter; lard; sugar and honey.
Group 3: Processed foods	Jam; cured traditional ham; olives; canned fruits; salted or sugared nuts; canned or bottled vegetables and legumes; breads; artisanal pizza; smoked and canned fish; cheese; wine and beer.
Group 4: Ultra-processed food	Processed meat (e.g., salami, mortadella, sausages, hamburger, chicken nuggets); fish products (e.g., fish sticks); packaged breads and buns; bread substitutes (e.g., crackers, rusks, breadstick); breakfast cereals and bars; fruit yogurt; fruit drinks; carbonated soft drinks; cocoa drinks; alcoholic drinks (e.g., rum, gin, whisky); energy drinks and bars; milk substitutes (e.g., soy drinks); margarine; mayonnaise and similar; sliced cheese; sweet packaged snacks; plant-based meat alternatives (e.g., veggie burgers); non-sugar sweeteners; sweet biscuits; cakes, croissant and other non-handmade pastries; ice-cream; chocolate; candies and gums; non-sugar sweeteners; baby food.

**Table 2:** Characteristics of 8688 participants (20–97 years) in the INHES study, Italy, 2010–2013.

		Meal Timing Pattern		
	All	Early Eaters	Late Eaters	*p*-Value
N of subjects, %	8688 (100.0)	5781 (66.5)	2907 (33.5)	-
Sex				0.44
Men	4053 (46.7)	2680 (46.4)	1373 (47.3)	
Women	4635 (53.3)	3101 (53.6)	1534 (52.8)	
Age (years; mean ± SD)	56.9 ± 14.6	58.9 ± 14.5	52.9 ± 13.9	<0.0001
Age groups, years				<0.0001
19–50	2967 (34.2)	1718 (29.7)	1249 (43.0)	
51–65	2863 (32.9)	1799 (31.1)	1064 (36.6)	
66–97	2858 (32.9)	2264 (39.2)	594 (20.4)	
Geographical area				<0.0001
Northern	3556 (40.9)	2932 (50.7)	624 (21.5)	
Centre	1407 (16.2)	886 (15.3)	521 (17.9)	
Southern	3725 (42.9)	1963 (34.0)	1762 (60.6)	
Place of residence				<0.0001
Rural	1178 (13.6)	861 (14.9)	317 (10.9)	
Urban	7510 (86.4)	4920 (85.1)	2590 (89.1)	
Educational level				<0.0001
Up to elementary	1540 (17.7)	1252 (21.7)	288 (9.9)	
Lower secondary	2268 (26.1)	1589 (27.5)	679 (23.4)	
Upper secondary	3430 (39.5)	2142 (37.0)	1288 (44.3)	
Postsecondary	1385 (15.9)	747 (12.9)	638 (21.9)	
Missing data	65 (0.8)	51 (0.9)	14 (0.5)	
Occupation				0.0001
Non-manual workers	2658 (30.6)	1525 (26.4)	1133 (39.0)	
Manual workers	1537 (17.7)	1006 (17.4)	531 (18.3)	
Housewife	958 (11.0)	623 (10.9)	325 (11.2)	
Retired	3129 (36.0)	2406 (41.6)	723 (24.9)	
Student	142 (1.6)	61 (1.1)	81 (2.7)	
Unemployed	251 (2.9)	145 (2.5)	106 (3.6)	
Missing data	13 (0.2)	5 (0.1)	8 (0.3)	
Marital status				0.19
Married/in couple	6533 (75.2)	4382 (75.8)	2151 (74.0)	
Single	1244 (14.3)	707 (12.2)	537 (18.5)	
Separated/divorced	270 (3.1)	185 (3.2)	85 (2.9)	
Widowed	616 (7.1)	492 (8.5)	124 (4.3)	
Missing data	25 (0.3)	15 (0.3)	10 (0.3)	
Smoking habit				<0.0001
No	5180 (59.6)	3533 (61.1)	1647 (56.7)	
Current	1390 (16.0)	888 (15.4)	502 (17.3)	
Ex	1925 (22.2)	1244 (21.5)	681 (23.4)	
Occasional	163 (1.9)	96 (1.7)	67 (2.3)	
Missing data	30 (0.3)	20 (0.3)	10 (0.3)	
Sport activity				0.067
No	7096 (81.7)	4835 (83.6)	2261 (77.8)	
Yes	1585 (18.2)	943 (16.3)	642 (22.1)	
Missing data	7 (0.1)	3 (0.1)	4 (0.1)	
Cardiovascular disease				0.095
No	8397 (96.7)	5576 (96.6)	2821 (97.0)	
Yes	291 (3.3)	205 (3.4)	86 (3.0)	
Cancer				0.73
No	8397 (96.6)	5570 (96.3)	2827 (97.2)	
Yes	291 (3.4)	211 (3.7)	80 (2.8)	
Hypertension				0.026
No	5859 (67.4)	3762 (65.1)	2097 (72.1)	
Yes	2809 (32.4)	2008 (34.7)	801 (27.6)	
Missing data	20 (0.2)	11 (0.2)	9 (0.3)	
Hyperlipidaemia				0.010
No	6756 (77.8)	4456 (77.2)	2300 (79.0)	
Yes	1902 (21.9)	1307 (22.5)	595 (20.6)	
Missing data	30 (0.3)	18 (0.3)	12 (0.4)	
Diabetes				0.33
No	7997 (92.1)	5281 (91.4)	2716 (93.4)	
Yes	661 (7.6)	482 (8.3)	179 (6.2)	
Missing data	30 (0.3)	18 (0.3)	12 (0.4)	
Body mass index				0.13
Normal weight	4168 (48.0)	2727 (47.2)	1441 (49.6)	
Overweight	3333 (38.3)	2250 (38.9)	1083 (37.2)	
Obese	1172 (13.5)	795 (13.8)	377 (13.0)	
Missing data	15 (0.2)	9 (0.1)	6 (0.2)	

Values are reported as numbers and percentages unless otherwise stated. Means were adjusted for age, sex and energy intake. *p*-values were obtained using generalized linear models for both continuous and categorical dependent variables adjusted for age, sex and energy intake.

**Table 3:** Dietary factors associated with meal timing pattern in 8688 participants (20–97 years) from the INHES study, Italy, 2010–2013.

	Meal Timing Pattern		
	Early Eaters	Late Eaters	*p*-Value
Energy intake (kcal/d)	1889 ± 578	1913 ± 592	0.093
Alcohol intake (g/d)	9.0 ± 14.5	8.9 ± 13.9	0.87
Carbohydrate (% TEI)	49.1 ± 9.9	48.5 ± 9.7	0.018
Sugar (g/d)	70.1 ± 29.9	69.4 ± 30.2	0.30
Fibre intake (g/d)	18.0 ± 7.8	18.1 ± 8.1	0.48
Protein (% TEI)	16.0 ± 3.8	16.1 ± 3.8	0.27
Fat (% TEI)	34.6 ± 7.9	35.1 ± 7.8	0.0079
Saturated fat (% TEI)	10.1 ± 3.8	10.3 ± 3.7	0.085
Saturated fat (g/d)	21.6 ± 11.4	21.9 ± 11.5	0.11
MUFA (% TEI)	10.1 ± 3.8	10.3 ± 3.7	0.085
PUFA (% TEI)	4.2 ± 1.6	4.2 ± 1.6	0.26
Dietary cholesterol (mg/d)	235.7 ± 168.4	232.1 ± 168.9	0.34
Sodium (mg/d)	1620 ± 1095	1600 ± 1063	0.36
Minimally processed food (Group 1)	74.0 ± 11.8	72.7 ± 12.2	<0.0001
Culinary ingredients (Group 2)	2.6 ±1.2	2.6 ± 1.2	0.12
Processed food (Group 3)	15.9 ± 10.6	16.4 ± 10.7	0.033
Ultra-processed food (Group 4)	7.5 ± 6.7	8.3 ± 7.3	<0.0001

TEI = total energy intake. MUFA = monounsaturated fats. PUFA = polyunsaturated fats. Means and *p*-values obtained from general linear regression models adjusted for sex, age and energy intake.

**Table 4:** Association of food processing according to NOVA classification with meal timing pattern in 8688 participants (20–97 years) from the INHES study, Italy 2010–2013.

	Meal Timing Pattern
	Late vs. Early Eaters
NOVA Groups	β (95% CI)
Minimally processed food (Group 1)	
Model 1	−0.11 (−0.15 to −0.07)
Model 2	−0.10 (−0.14 to −0.06)
Culinary ingredients (Group 2)	
Model 1	0.03 (−0.01 to 0.08)
Model 2	0.05 (0.01 to 0.10)
Processed food (Group 3)	
Model 1	0.04 (0.003 to 0.08)
Model 2	0.02 (−0.02 to 0.06)
Ultra-processed food (Group 4)	
Model 1	0.11 (0.07 to 0.15)
Model 2	0.13 (0.09 to 0.18)
Mediterranean Diet Score	
Model 1	−0.03 (−0.07 to 0.01)
Model 2	−0.07 (−0.12 to −0.03)
FSAm-NPS dietary index	
Model 1	0.05 (0.01 to 0.10)
Model 2	0.10 (0.05 to 0.14)

Model 1: Multivariable-adjusted linear regression including age, sex and energy intake. Model 2: Multivariable-adjusted linear regression including age, sex, energy intake, place of residence, educational level, occupation, marital status, smoking status, sport activity, body mass index, history of cardiovascular disease, history of cancer, diabetes, hyperlipidaemia and hypertension. FSAm-NPS = Food Standards Agency Nutrient Profiling System. Each dietary variable was standardized to allow comparison.

## Data Availability

The data underlying this article will be shared on reasonable request to the corresponding author. The data are stored in an institutional repository (https://repository.neuromed.it) and access is restricted by ethical approval and the legislation of the European Union.

## Supplementary Materials

The following supporting information can be downloaded at https://www.mdpi.com/article/10.3390/nu15061497/s1. Table S1: Association of food processing according to NOVA classification with meal timing pattern across age groups from the INHES Study, Italy 2010–2013; Table S2: Association of food processing according to NOVA classification with meal timing pattern in men and women from the INHES Study, Italy 2010–2013.

## Appendix A

**INHES Study Investigators**

Principal Investigator: Licia Iacoviello

Study coordinator: Americo Bonanni

**Scientific Committee:** Marialaura Bonaccio, Francesca Bracone, Chiara Cerletti, Simona Costanzo, Augusto Di Castelnuovo, Mariarosaria Persichillo, Maria Benedetta Donati, Giovanni de Gaetano.

**Dietary questionnaire validation**: Mariarosaria Persichillo and Francesco Zito.

**Questionnaire administration:** Lucia Aurisano, Paola Barisciano, Valentina Bonaccio, Francesca De Lucia, Giovanna Galuppo, Filippo Petrucci, Anna Sciarretta, Angelita Verna.

**Data management:** Simona Costanzo, Augusto Di Castelnuovo, Marco Olivieri.
