# Supplementary material for: Association between Late-Eating Pattern and Higher Consumption of Ultra-Processed Food among Italian Adults: Findings from the INHES Study
Source: Nutrients. 2023 Mar 20;15(6):1497. doi: 10.3390/nu15061497 (PMC10058735; doi:10.3390/nu15061497)
Supplement: Supplementary file 1 [file nutrients-15-01497-s001.zip › nutrients-2227221-supplementary.pdf]

nutrients

MDPI

Supplementary Materials

A Late Eating Pattern is Associated with Higher Consumption of Ultra-processed Food among Italian Adults from the INHES Study

Marialaura Bonaccio [1,*], Emilia Ruggiero [1], Augusto Di Castelnuovo [2], Claudia Francisca Martínez [1], Simona Esposito [1], Simona Costanzo [1], Chiara Cerletti [1], Maria Benedetta Donati [1], Giovanni de Gaetano [1] and Licia Iacoviello [1,3] for the INHES Study Investigators [†]

Table S1. Association of food processing according to NOVA classification with meal timing pattern across age groups from the INHES Study, Italy 2010–2013.

	Meal timing pattern (Late vs. early eaters)			
	**Aged 19-50 y (n=2967; 34.2%)**	**Aged 51-65 y (n=2863; 32.9%)**	**Aged 66-97 y (n=2858; 32.9%)**	**P value for interaction**
**NOVA groups**	**β (95%CI)**	**β (95%CI)**	**β (95%CI)**	
Minimally processed food (Group 1)	-0.17 (-0.24 to -0.10)	-0.09 (-0.16 to -0.02)	0.01 (-0.08 to 0.09)	0.0074
Culinary ingredients (Group 2)	0.01 (-0.07 to 0.08)	0.04 (-0.04 to 0.12)	0.12 (0.03 to 0.21)	0.094
Processed food (Group 3)	0.10 (0.03 to 0.16)	-0.01 (-0.09 to 0.06)	-0.07 (-0.16 to 0.01)	0.0042
Ultra-processed food (Group 4)	0.13 (0.05 to 0.22)	0.17 (0.10 to 0.25)	0.07 (-0.01 to 0.15)	0.59
Mediterranean Diet Score	-0.07 (-0.14 to 0.002)	-0.12 (-0.19 to -0.05)	-0.03 (-0.12 to 0.05)	0.37
FSAm-NPS dietary index	0.07 (-0.01 to 0.15)	0.14 (0.06 to 0.22)	0.09 (0.004 to 0.18)	0.40

Regression coefficients with 95% confidence intervals obtained from a multivariable-adjusted linear regression including age, sex, energy intake, place of residence, educational level, occupation, marital status, smoking status, sport activity, body mass index, history of cardiovascular disease, history of cancer, diabetes, hyperlipidaemia and hypertension.

FSAm-NPS = Food Standards Agency Nutrient Profiling System.

Each dietary variable was standardized to allow comparison.

Table S2. Association of food processing according to NOVA classification with meal timing pattern in men and women from the INHES Study, Italy 2010–2013.

	**Meal timing pattern (Late vs. early eaters)**		
	**Men (n=4053; 46.6%)**	**Women (n=4635; 53.4%)**	**P value for interaction**
**NOVA groups**	**β (95%CI)**	**β (95%CI)**	
Minimally processed food (Group 1)	-0.08 (-0.15 to -0.01)	-0.13 (-0.18 to -0.08)	0.045
Culinary ingredients (Group 2)	-0.01 (-0.08 to 0.06)	0.10 (0.03 to 0.16)	0.0016
Processed food (Group 3)	0.02 (-0.05 to 0.09)	0.03 (-0.02 to 0.08)	0.29
Ultra-processed food (Group 4)	0.11 (0.04 to 0.18)	0.15 (0.09 to 0.22)	0.25
Mediterranean Diet Score	-0.07 (-0.13 to -0.01)	-0.08 (-0.14 to -0.02)	0.71
FSAm-NPS dietary index	0.10 (0.03 to 0.16)	0.10 (0.03 to 0.16)	0.96

Regression coefficients with 95% confidence intervals obtained from a multivariable-adjusted linear regression including age, energy intake, place of residence, educational level, occupation, marital status, smoking status, sport activity, body mass index, history of cardiovascular disease, history of cancer, diabetes, hyperlipidaemia and hypertension.

FSAm-NPS = Food Standards Agency Nutrient Profiling System.

Each dietary variable was standardized to allow comparison.
